# Iatrogenic incarcerated diaphragmatic hernia following laparoscopic resection of a diaphragmatic cystic lymphangioma: A case report

**DOI:** 10.1016/j.ijscr.2023.108947

**Published:** 2023-10-10

**Authors:** Anis Hasnaoui, Racem Trigui, Sihem Heni, Prakash V.A.K. Ramdass

**Affiliations:** a Faculty of Medicine of Tunis, Tunis El Manar University, Department of General Surgery, Menzel Bourguiba Hospital, Rue Djebal Lakhdar, 1006 Tunis, Tunisia; b St. George's University School of Medicine, Department of Public Health and Preventive Medicine, St. George, Grenada

**Keywords:** Diaphragmatic hernia, Iatrogenic disease, Intestinal obstruction, Emergencies, Case report

## Abstract

**Introduction:**

Incarcerated iatrogenic right diaphragm hernia stands as a rare occurrence. Swift diagnosis and timely management are imperative. This article presents a particularly uncommon case of a right diaphragmatic hernia resulting from a neglected defect following the resection of a diaphragmatic lymphangioma and sheds light on the pitfalls that may lead to such a post-operative event.

**Presentation of case:**

Our surgical ward admitted a 36-year-old patient presenting symptoms indicative of bowel obstruction. Four months earlier, the patient had undergone laparoscopic resection of a lymphangioma located in the right dome of the diaphragm. Thoracic auscultation yielded hyperactive bowel sounds at the lower lung field and the right costophrenic angle. Abdominal distention was observed without any signs of peritoneal irritation. A thoracic and abdominal CT scan provided confirmation of a right diaphragmatic hernia. Subsequently, an immediate laparotomy was performed. The right colic hepatic flexure was released, and the diaphragmatic breach was sutured. The postoperative course was uneventful.

**Discussion:**

Iatrogenic diaphragmatic hernia remains a possible complication of this surgery. It could occur even on the right side where the liver has a cushioning effect. Incarcerated diaphragmatic hernia is a surgical emergency that should be operated on as quickly as possible. A systematic check of the diaphragm's integrity at the end of the surgical procedure could prevent such complications.

**Conclusion:**

While surgical techniques and laparoscopic instrumentation have witnessed significant advancements in recent years, achieving proficiency and the precise execution of surgical techniques remain of utmost importance.

## Highlights

• Iatrogenic diaphragmatic hernia remains a possible complication after diaphragmatic surgery.
• Diaphragmatic hernia could occur on the right side even with the cushioning effect of the liver.
• Incarceration of a bowel in a diaphragmatic hernia could be life-threatening.
• A systematic check of the diaphragm is mandatory after liver or diaphragmatic surgeries.

## 1 Introduction

In contemporary surgery, laparoscopy has emerged as a prominent approach for both the diagnosis and treatment of gastrointestinal neoplasms. Vigilance towards all potential risks, regardless of their infrequency, is paramount. Among the spectrum of complications, iatrogenic diaphragmatic hernia stands out as an exceptionally rare and distinctive occurrence subsequent to laparoscopic diaphragmatic and hepatic surgeries [1,2]. The existing body of literature addressing this phenomenon is notably limited, primarily comprising sporadic case reports. This underscores the pressing need for a more comprehensive understanding and heightened awareness of this rare but significant surgical event. This article presents a case of a complicated right diaphragmatic hernia resulting from a neglected defect following the resection of a diaphragmatic lymphangioma and sheds light on the pitfalls that may lead to such a post-operative event. This work has been reported in line with the SCARE criteria [3].

**Fig. 1:**
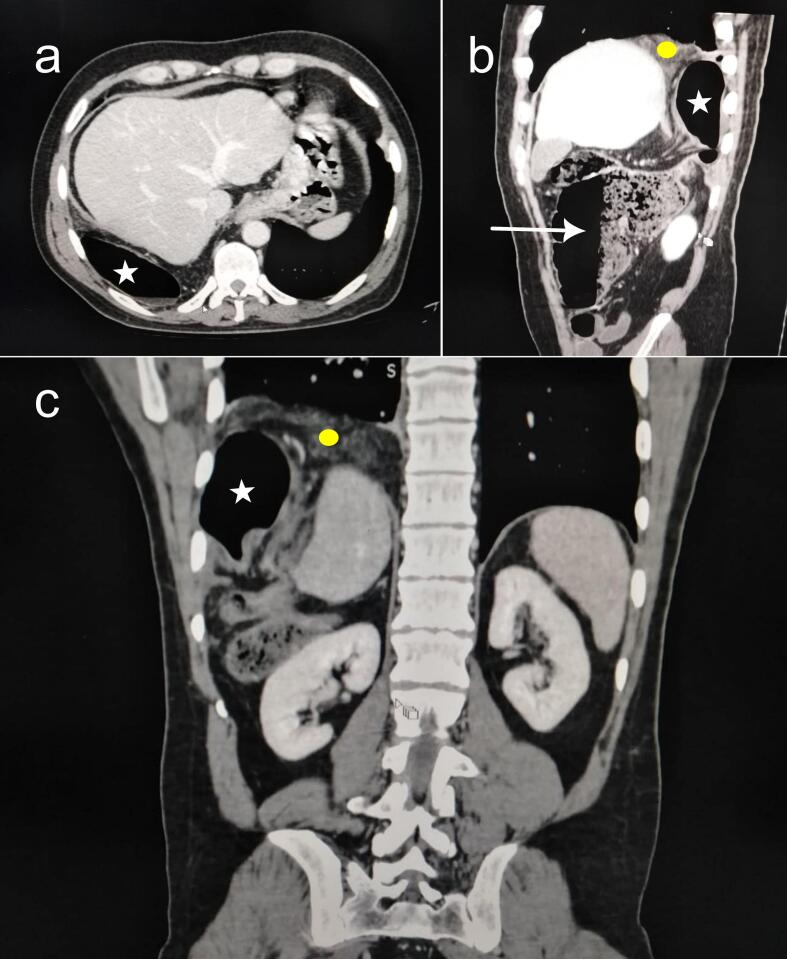
Preoperative CT scan. Portal phase (a) axial view, (b) sagittal view, and coronal view showing the incarcerated right colic flexure (white stars) and omentum (yellow circles). The ascending colon (white arrow) is distended as shown in (b). (For interpretation of the references to colour in this figure legend, the reader is referred to the web version of this article.)

## 2 Presentation of case

**Fig. 2:**
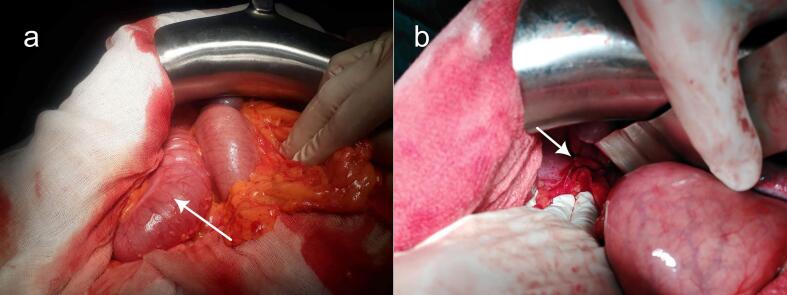
Intraoperative view before the release of the strangulation. (a) The right colic flexure diving beneath the liver and into the thorax with upstream distension of the ascending colon (white arrow). (b) White arrow pointing to the diaphragmatic defect.

**Fig. 3:**
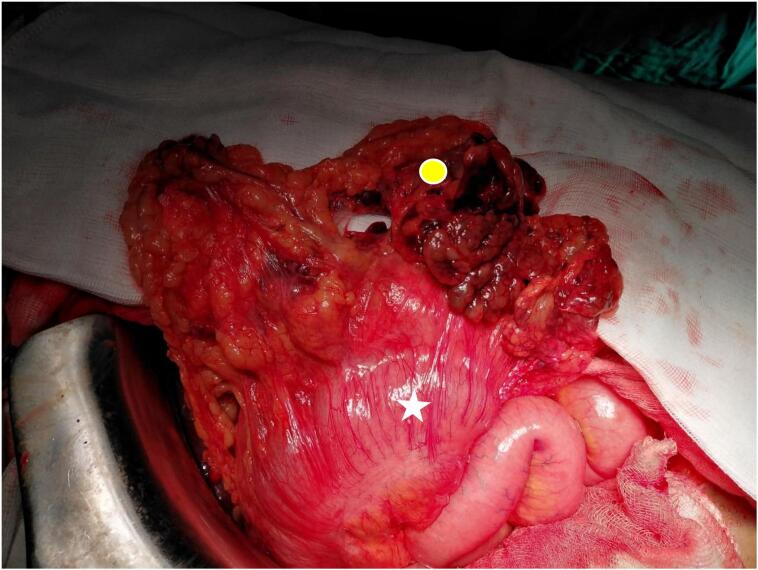
Intraoperative view after the release of the strangulation. Notice the congestive colon (white star) and the omentum containing areas of necrosis (yellow circle). (For interpretation of the references to colour in this figure legend, the reader is referred to the web version of this article.)

A 36-year-old patient was admitted to our surgery ward with symptoms indicative of bowel obstruction that had been progressing for 24 h. These included abdominal pain, constipation, an inability to pass gas, and mild abdominal distension. Four months earlier, the patient had undergone laparoscopic abdominal surgery to excise a cystic lymphangioma located in the right dome of the diaphragm at a different hospital. According to the patient, the immediate postoperative period had been without any noteworthy events. Upon examination, the patient was conscious, afebrile, with a blood pressure reading of 130/70 mmHg, and a heart rate of 95 beats per minute. Respiratory assessment revealed effortless breathing characterized by a regular pattern and a rate of 15 breaths per minute. Thoracic auscultation yielded hyperactive bowel sounds at the right lower lung field and the right costophrenic angle. Abdominal examination unveiled distension without signs of peritoneal irritation. No incisional or ventral hernias were observed. The rectal examination did not reveal any masses, bleeding, or fecal matter. Laboratory findings displayed an elevated white blood cell count, but there were no renal function impairment or electrolyte disorders. An emergency thoracic and abdominal CT scan was conducted, conclusively confirming the diagnosis of a right diaphragmatic hernia with a 5-centimeter defect. Through this opening, the right colic flexure and its mesentery had ascended into the chest cavity, leading to distension of the ascending colon (Fig. 1). Following a brief resuscitation, the patient underwent an emergency laparotomy. There was distension of the ascending colon upstream of an incarceration of the right colic flexure in a 5 cm diaphragmatic defect (Fig. 2). The section of the fibrous ring of the diaphragm allowed for the release of a congestive colon and the greater omentum containing some areas of necrosis (Fig. 3). Following a thorough peritoneal lavage, the diaphragmatic breach was meticulously sutured using non-absorbable interrupted sutures, and no drainage was deemed necessary. The leak test was negative. An immediate postoperative chest radiograph was performed, revealing a slight blunting of the right costophrenic angle but no pneumothorax was detected. The post-operative course was uneventful, and the patient was discharged four days later. Six months after surgery, the patient was symptom-free. Clinical examination and chest radiograph were normal.

## 3 Discussion

Although laparoscopic excision of lesions on the diaphragm has been widely utilized and standardized, iatrogenic diaphragmatic hernia remains a possible complication of this surgery with an incidence yet to be determined [4]. To our knowledge, this report represents the first documented case of an iatrogenic diaphragmatic hernia following laparoscopic resection of a cystic lymphangioma. Iatrogenic injuries are considered an uncommon cause of diaphragmatic hernias, resulting in the protrusion of abdominal viscera or omentum into the chest cavity, potentially leading to a life-threatening situation if left untreated [5]. The right hemidiaphragm, due to the cushioning effect of the liver, is generally more protected than the left. Consequently, right diaphragmatic ruptures typically result from direct penetrating trauma or intraoperative injuries [6]. Through the literature survey, most of the iatrogenic right diaphragmatic hernias are illustrated in scattered case reports following laparoscopic cholecystectomy [7], laparoscopic fenestration of benign cysts [1], hepatic resection [2], right nephrectomy [8] and accounting only for 52 cases over a period of 24 years. Because of these rather limited scientific publications on the subject, its exact incidence, mechanisms, and prevention are still mysteries that elude us to the present day.

In a recent study conducted on incarcerated diaphragmatic hernia after right hepatectomy, published by Won Lee and al. in 2021 [9], risk factors for developing iatrogenic diaphragm rapture have been identified, including the volume of the resected liver, thermal injury, direct trauma, the patient's nutritional status, developing ascites in the postoperative course, a high-pressure gradient through the diaphragm, and the quality of the diaphragm tissue. Other reports suggested that high-energy instruments, such as the ultrasonic activated scissor, are responsible for diaphragm injuries [10]. The most underrated risk factor in all previously published reports is the surgeon's experience: injuries can occur due to careless management of instruments, thermofusion, and the ultrasonic dissector [7,11]. Although supervision has increased in operating rooms, we believe that even a small accidental injury to the diaphragm due to a technical fault, compounded by the thoraco-abdominal pressure gradient, can be responsible for diaphragmatic rupture [12]. With the rising number of laparoscopic surgeries performed, a surgeon's learning curve increases over time, making him aware of pitfalls and technical errors that might occur. Checking the diaphragm's integrity after a close-contact surgery, especially if using high-energy instruments is mandatory, and a lesson yet to be taught to younger and more inexperienced surgeons [13]. Therefore, surgeries including right liver mobilization, and diaphragm close dissection should be reserved for senior surgeons or supervised juniors, to limit this unexpected and fatal complication [14].

Clinical features mainly depend on the size of the diaphragm defect; in the case of large defects, early symptoms are to be expected, essentially hemodynamic and respiratory instabilities. Like in our case, when a diaphragmatic hernia is of late onset, the clinical picture is dominated by chest and abdominal pain, nausea, and vomiting, with the possibility of evolving into bowel obstruction if intestinal loops are incarcerated [1]. Several imaging techniques are employed to achieve a correct diagnosis. Chest X-ray is a useful first-intention screening test, but CT scan continues to be the imaging modality of choice for a positive diagnosis. Despite its high sensitivity, the specificity of a CT scan is limited to 50 % [15]. Considering the fast-settling complications (ischemia and bowel necrosis), an iatrogenic incarcerated diaphragmatic hernia is a surgical emergency that should be operated on as quickly as possible. To appropriately manage abdominal organs, laparotomy is advised. However, in cases of delayed iatrogenic incarcerated diaphragmatic hernias, to prevent fatal outcomes due to intra-thoracic visceral perforation, an adjoining thoracotomy is recommended [6]. Although the most efficient technique to ensure closure of a diaphragmatic defect is still a subject of controversy, most authors agreed that these defects can be safely repaired using only non-absorbable sutures without requiring the use of a prosthetic mesh [16,17]. In our case, the diaphragm defect was closed with non-absorbable sutures, with a negative leak test at the end of the procedure. This procedure involves immersing the repair in a saline solution while deliberately raising intra-thoracic pressure. A positive test will reveal air bubbles, signifying an insufficient repair that necessitates revision.

## 4 Conclusion

Iatrogenic herniation of the abdominal viscera after laparoscopic surgery is an unfortunate post-operative event. Such complications can be prevented by close supervision in the operating rooms by senior surgeons, respecting the learning curve, careful surgical dissection, correct management of high-energy instruments, and above all, a systematic check of the diaphragm's integrity at the end of the surgical procedure.

## Consent for publication

A written consent was obtained from the patient to publish this case report.

## Ethical approval

Ethical approval was deemed unnecessary by our institutional ethical committee, as the paper is reporting a single case that emerged during normal practice.

## Funding

This research did not receive any specific grant from funding agencies in the public, commercial, or not-for-profit sectors.

## Author contribution

Anis Hasnaoui: Conceptualization, Writing-Reviewing and Editing. Racem Trigui: writing-Original draft preparation. Sihem Heni: Data curation. Prakash V.A.K. Ramdass: Writing-Reviewing. All authors read and approved the final manuscript.

## Guarantor

Anis Hasnaoui

## Conflict of interest statement

The authors declare that they have no competing interests.

## References

[1] Soufi M., Meillat H., Le Treut Y.-P. (2013). Right diaphragmatic iatrogenic hernia after laparoscopic fenestration of a liver cyst: report of a case and review of the literature. World J. Emerg. Surg..

[2] Hawxby A.M., Mason D.P., Klein A.S. (2006). Diaphragmatic hernia after right donor and hepatectomy: a rare donor complication of partial hepatectomy for transplantation. Hepatobiliary Pancreat. Dis. Int..

[3] Agha R.A., Franchi T., Sohrabi C., Mathew G., Kerwan A., Thoma A., Beamish A.J., Noureldin A., Rao A., Vasudevan B., Challacombe B., Perakath B., Kirshtein B., Ekser B., Pramesh C.S., Laskin D.M., Machado-Aranda D., Miguel D., Pagano D., Millham F.H., Roy G., Kadioglu H., Nixon I.J., Mukherjee I., McCaul J.A., Chi-Yong Ngu J., Albrecht J., Rivas J.G., Raveendran K., Derbyshire L., Ather M.H., Thorat M.A., Valmasoni M., Bashashati M., Chalkoo M., Teo N.Z., Raison N., Muensterer O.J., Bradley P.J., Goel P., Pai P.S., Afifi R.Y., Rosin R.D., Coppola R., Klappenbach R., Wynn R., De Wilde R.L., Surani S., Giordano S., Massarut S., Raja S.G., Basu S., Enam S.A., Manning T.G., Cross T., Karanth V.K.L., Kasivisvanathan V., Mei Z., The S.C.A.R.E. (2020). Guideline: updating consensus Surgical CAse REport (SCARE) guidelines. Int. J. Surg..

[4] Hashimoto K., Obama K., Tsunoda S., Hisamori S., Nishigori T., Sakaguchi M., Ueda Y., Nakanishi N., Sakai Y. (2020). Iatrogenic diaphragmatic hernia as a late complication of laparoscopic excisional biopsy of peritoneal nodules: a case report. Int. J. Surg. Case Rep..

[5] Crandall M., Popowich D., Shapiro M., West M. (2007). Posttraumatic hernias: historical overview and review of the literature. Am. Surg..

[6] Testini M., Girardi A., Isernia R.M., De Palma A., Catalano G., Pezzolla A., Gurrado A. (2017). Emergency surgery due to diaphragmatic hernia: case series and review. World J. Emerg. Surg..

[7] Armstrong P.A., Miller S.F., Brown G.R. (1999). Diaphragmatic hernia seen as a late complication of laparoscopic cholecystectomy. Surg. Endosc..

[8] Mínguez Ruiz G., García Florez L.J., Arias Pacheco R.D., García Bear I., Ramos Pérez V., Pire Abaitua G. (2018). Post-nephrectomy diaphragmatic hernia. Increase suspicion and decrease morbi-mortality: two cases report. J. Surg. Case Rep..

[9] Lee S.W., Lee S.D., Kim M.-Y. (2021). Incarcerated diaphragmatic hernia after right hepatectomy: an autopsy case with a review of 45 previous cases. Int. J. Legal Med..

[10] Suh Y., Lee J.H., Jeon H., Kim D., Kim W. (2012). Late onset iatrogenic diaphragmatic hernia after laparoscopy-assisted total gastrectomy for gastric cancer. J. Gastric Cancer.

[11] de Meijer V.E., Vles W.J., Kats E., den Hoed P.T. (2008). Iatrogenic diaphragmatic hernia complicating nephrectomy: top-down or bottom-up?. Hernia J. Hernias Abdom. Wall Surg..

[12] Watkins A.A., Kalluri A., Gupta A., Gangadharan S.P. (2021). Iatrogenic diaphragmatic hernia with fecopneumothorax following minimally invasive esophagectomy and liver resection. JTCVS Tech..

[13] Potter S.R., Kavoussi L.R., Jackman S.V. (2001). Management of diaphragmatic injury during laparoscopic nephrectomy. J. Urol..

[14] Addeo P., Schaaf C., Noblet V., Faitot F., Lebas B., Mahoudeau G., Besch C., Serfaty L., Bachellier P. (2021). The learning curve for piggyback liver transplantation: identifying factors challenging surgery. Surgery..

[15] Sandstrom C.K., Stern E.J. (2011). Diaphragmatic hernias: a spectrum of radiographic appearances. Curr. Probl. Diagn. Radiol..

[16] Guner A., Ozkan O.F., Bekar Y., Kece C., Kaya U., Reis E. (2012). Management of delayed presentation of a right-side traumatic diaphragmatic rupture. World J. Surg..

[17] Soper N.J., Teitelbaum E.N. (2013). Laparoscopic paraesophageal hernia repair: current controversies. Surg. Laparosc. Endosc. Percutan. Tech..

